# Twelve tips for Applying to Clinical Academic Training in the United Kingdom

**DOI:** 10.15694/mep.2019.000053.1

**Published:** 2019-03-13

**Authors:** Mohammed Abdul Waduud, Nadeem Ahmed, Julian Scott

**Affiliations:** 1 University of Leeds; 2 University of Birmingham; 3 Leeds Teaching Hospitals NHS Trust

**Keywords:** Integrated Academic Training, United Kingdom, Tips

## Abstract

This article was migrated. The article was marked as recommended.

MAW and colleagues offer their advice on applying for academic clinical training posts including the do’s and don’ts. The authors all have experience of the national Integrated Academic Training (IAT) pathway in the United Kingdom. Whilst all the following top tips are not mandatory to attain a clinical academic role, we believe they would put a potential applicant in a good position to succeed, regardless of whether they were applying for an academic foundation post, academic clinical fellowship or a clinical lectureship. We have tailored our advice so that it may be considered when constructing an application as well as helping applicants for the interview.

## Introduction

In the United Kingdom (UK), academic clinical trainees account for approximately 5% of the medical workforce [ Clough, 2017]. Importantly, training pathways vary in England (National Institute for Health Research [NIHR] Integrated Academic Training (IAT) Programme), Scotland (Scottish Academic Training [SCREDS]) and Wales (Welsh Clinical Academic Track [WCAT]) [ National Institute for Health Research, 2019][ Scottish Medical Training, 2013][ Health Education and Improvement Wales, 2018]. Regardless of the national geographic variations in training the majority of the pathways involve a form of academic foundation training, a pre-doctoral academic fellowship (for example a NIHR ACF) and post-doctoral academic Clinical Lectureship (CL). All schemes are orientated to produce competitive candidates who are able to apply for fellowship funding at doctoral (PhD) level or, at a more senior level, intermediate/advanced fellowships or tenure track academic posts at University or other Higher Education Institutions (HEIs).

Academic posts have both a clinical and academic elements. The proportion of protected research time offered with academic training posts can differ across the UK. Commonly academic foundation posts have a 4 month research rotation within the two year foundation post, ACFs have 25% full-time equivalent (FTE) academic research time over 3 years and CL have a 50% FTE academic research time for up to 4 years until CCT (Certificate of Completion of Training) in England or 20% FTE academic research time for up to 6 years in Scotland and Wales [ National Institute for Health Research, 2019][ Scottish Medical Training, 2013][ Health Education and Improvement Wales, 2018]. Academic posts are also linked to an academic National Training Number (NTNa) and, as for all clinical trainees, postholders have an annual appraisal which assesses their clinical competencies; in addition academic trainees are also assessed on the academic component of their training [ Academy for Medical Royal Colleges, 2017]. It is important to consider these expectations prior to a prospective application as you would be expected to achieve certain academic competencies in addition to your clinical competencies.

The application process differs dependent on the type and location of the post. Academic foundation posts are managed nationally through the Oriel online national recruitment system, as are ACF and some CL posts in England [ Oriel, 2019]. Recruitment for the CL posts in Scotland, Wales and certain parts of England are, however, managed directly by the University within which the position is available [ Scottish Medical Training, 2013]. In terms of Oriel-based applications once registered on the system there will be available a number of posts at varying entry levels in a range of specialties; adverts for posts are normally disseminated at set times throughout the academic year (August for AFP and October for Round 1 ACF). CL posts are advertised throughout the year which may be identified through the host University’s website.

All ACFs and CLs are specialty specific. Each year, IAT partnerships are informed of the number of posts allocated by formula and invited to propose their preferences for medical specialties based on academic strengths and ability to support each specialty clinically. Postgraduate Deans, Medical School Deans and IAT leads take collective responsibility for the specialty spread being proposed and are key in preparing the partnership bids for additional ACF and CL posts to support the following NIHR Priority Research Themes of Platform Science and Bioinformatics, Therapeutics or Clinical Pharmacology, Complex Health Needs in Age-Related and Chronic Disease, Dementia, Medical Education, Acute Care and Mental Health.

Here we present top tips for the academic part of the application and interview which may pay dividends when applying for any form of clinical academic training post. Through our experience, we believe the following tips to be helpful in preparing for the application and the interview process.

## Tip 1. Fail to prepare, prepare to fail!

Know the job specification. Spend time understanding the job criteria as this will outline the minimum bench-mark you must meet in order to be successful for interview longlisting. Specifications for the vacancy you are applying to can be found on Oriel or the host deanery and/or University website. Seeking advice from senior colleagues may also help to decide if this is the right career choice for you.

Initiate contact with senior researchers at the host institution you are applying to by expressing your interest in applying for the advertised vacancy. This may provide you with a greater insight into the academic and clinical focuses of the host organisation, potential opportunities available and enable you to make an informed decision. It may also be valuable in planning your future career. If you have more time, then try to engage in academic work prior to applying for the post. This will demonstrate higher academic skills such as proactive organisation in initiating research design, design implantation and the ability to collaborate with colleagues.

Familiarise yourself with key researchers at the unit, identify specific fields they are interested in, and read key papers published by the unit as it shows you have done your homework and are serious about the job. Conversely if you are thinking of pursuing research that is not aligned at the institution of interest, knowledge of how you alternatively plan to achieve this is important. It can often be difficult to find a senior researcher who is willing to take on a different research project outside their topic of interest, but not impossible.

## Tip 2. Have a career goal in mind and have a plan on how you intend on achieving it

The classic interview question often revolves around the following question: “where do you see yourself in 5, 10, 20- or 30-years’ time?” Describe the clinical academic pathway in the region you are applying to and where you would fit in. Be prepared to explain how you aim to reach and accomplish each milestone rather than the idyllic academic position. An awareness of this pathway shows you have realistic expectations and are aware of the work involved! Whether you want to be a professor in your chosen speciality or simply develop your academic skills, have a vision you can sell. The institution may be willing to invest a lot of resources, time and money into you, tell them why it should be you.

## Tip 3. Pursue a Postgraduate Degree

For most candidates the initial exposure to academic clinical training may be as an undergraduate student on a specialist module, summer elective or an internal/external research undergraduate scholarship. In the UK, medical undergraduates may nurture an academic interest by extending their undergraduate medical training by an additional year, learning core research principles and conduct a piece of research via undertaking an intercalated bachelor’s degree. For those who are considering an academic career at an early stage of their training we would highly recommend undertaking an intercalated year within a group that has a proven track record of getting the undergraduate a national/international presentation and a paper. This may help to weigh up if an academic career is for you and develop some basic research skills which important for clinical training.

Nevertheless, if you have been unable to undertake an intercalated degree there are always other opportunities to undertake higher taught postgraduate degrees. Many universities across the UK now offer part-time certificates, diplomas and masters qualifications to medical graduates, as an alternative method of achieving a higher taught postgraduate degree. Some of these can be accessed as early as the first year of Foundation Training. Most academic foundation trainees and NIHR ACF are expected to complete a credit-bearing postgraduate qualification in an appropriate area of research training. Thus, higher qualifications may provide an additional source of evidence to augment your clinical learning, which can furthermore be mapped against components of the curriculum. Although an additional degree is not a prerequisite when applying for early academic training, candidates applying for an NIHR Clinical Lectureship must hold either an MD or PhD by the time of the interview.

## Tip 4. Sit your membership exams early

Certain membership colleges will let you sit your exams during your foundation training. For example, the MRCS examination could be started as during foundation year one and MRCP examination started during foundation year two [ Surgical Royal Colleges of the United Kingdom and in Ireland, 2018][ Royal College of Physicians, 2018]. Early preparation and sitting of membership exams can take the pressure off at later stages in your career, when you may accrue additional work-life commitments. Moreover, at some stage you in your academic training you will need to apply for a doctorate qualification (i.e. MD or PhD) and several major research funders often require postgraduate membership qualifications in order to apply for clinical research fellowships. Therefore having been successful in part 1 or 2 early is highly desirable and allows successful candidates the opportunity to focus on their research project, data collection and the formulation of PhD/MD proposal.

## Tip 5. Try to publish your work

Undergraduates and trainees often undertake a research or audit at some point in their career. However, most struggle to publish the research they have undertaken, often through no fault of their own. When undertaking projects, it is important to identify supervisors with a proven track record of published research, since you may not have the required experience to independently achieve a publication. Whether it is research, or a quality improvement project highlight your involvement in the task and describe how it has influenced clinical practice. Sometimes your work can lead to big changes in local healthcare policy so ask your past supervisors you have worked with whether your work has led to any changes.

Publications represent a hard outcome endpoint and are a significant achievement of your research. They highlight an array of research skills including study design, data analysis and manuscript writing. Moreover, publishing in a peer reviewed journal illustrates that you can produce work of a high standard approved by experts in the topic. Although authorship alone on a publication can be sufficient, be wary that some job applications may only recognise first, second or last author publications. At the early career level publications in peer-reviewed journals are well regarded regardless of the impact factor of the journal. However, when applying for senior positions such as lectureships the type of journal (i.e. generic versus specialist), impact factor and citations of published work become increasingly important as CLs can be returned in the REF (Research Excellence Framework).

## Tip 6. Be proactive and communicate your hard work

Presentations at conferences are an alternative method of demonstrating peer review and acceptance of your work. Attending conferences can also be a rewarding experience and can give you unique insight into your own work and the work of your colleagues. A simple discussion with a colleague whilst you present your poster may generate ideas you had not thought about before and help identify a direction to take your research. So take pride in your work and know it inside and out.

Furthermore, presentations also are awarded points during the academic application process and presentations at certain conferences can also lead to the publication of the abstract in associated specialist journals. However it can be a costly alternative. Conferences may be attended at a local, regional, national or international setting, with the later attracting a greater number of points. Be aware that there are generic and speciality specific conferences and the generalisability of your work should help determine which to target. Furthermore conferences may be targeted at an undergraduate or postgraduate audiences, therefore your academic experience and subject interest should help determine which is most appropriate for you.

We appreciate that prizes are difficult to achieve and often subject to tough competition. However, prizes do count for additional points during the application process. Common places to obtain prizes are conferences, as described above. Often there are prizes in poster categories as well as for oral presentations so when applying for a conference bare this in mind. In addition prizes may also be obtained through essay competitions that are advertised through the Royal Colleges and speciality societies.

Academics should also be well-rounded individuals with a good work-life balance. Not only is clinical and research excellence expected but individuals should be able to communicate their research in a wider setting. Research meetings and conferences along with newer online platforms including Twitter and networks such as researchgate.com, are becoming an increasing popular method of communicating amongst academics. Such encounters provide the opportunity to network with colleagues and learn about advancements within the field. If you’ve undertaken collaborative research or additional team activities in your own time highlight these in your application and interview.

## Tip 7. Follow the money

Whilst this seems unrelated and challenging to achieve there is truth in the old idiom, money makes the world go round. All academic institutions need to find ways to bring in money in the form of grants, bursaries or sponsors. This is often an important skill to develop as your training progresses and of particular relevance during the PhD or MD application, which is also in itself a request for funding by justifying your work to the funding body. Look for small pots of funding through summer student fellowships, internships, essay competitions, prizes and bursaries.

## Tip 8. Identify a few referees that will support your claims

Having a good supervisor and mentor can make all the difference with referees being academic or clinical supervisors. Have a couple of referees in mind who can provide references that vouch for both your clinical and academic integrity. Inform your referees of your intention to submit them as referees and approach your selected referees early with an up to date CV and your career intentions. No matter who your referees are, they will often have a wealth of experience they can impart throughout the application process. Whilst not essential try and have a referee in the specialty you are applying too.

## Tip 9. Remember, you are a clinician first and foremost

As doctors our duty first and foremost is to our patients. Early career academic clinicians have protected time allocated for academic research with the remaining time deemed clinical. Try to relate to how your academic endeavours are likely to support your clinical training as they often go hand in hand. It is important to show that you are a team player and unlikely to shift your clinical duties on to your colleagues in preference to your academic commitments. Conversely take pride in your academic commitments and try to convey the importance of your academic responsibilities to your clinical colleagues so they are aware. It is a delicate balance to maintain so have strategies rehearsed on how you may overcome these.

## Tip 10. Ensure you have some teaching experience

It’s not just about the research. Teaching experience when applying for clinical academic training can also go a long way. This is a separate branch of academia in its own right with the introduction of the recent Teaching Excellence Framework (TEF). The ability to teach undergraduates and colleagues is a skill which is becoming increasing important. Attempt to be involved and develop established programmes with evidence of good practice and feedback. If nothing exists you could try to organise one yourself, however ensure you have plenty of helpers so that it can be a success. Journal clubs as well as mentoring sessions can also count especially if conducted as a work-based assessment. No matter which avenue you pursue ensure you obtain feedback which can be quantified. Create your own feedback form or try to obtain one from a colleague. Record the number of students, their year of study and the subject taught. This will help demonstrate your ability to deliver quality teaching to a variety of audiences during the recruitment process.

## Tip 11. Remain calm during the dreaded interviews

The interview panel typically consists of university academics, clinicians from the health service (usually the lead for core training or speciality you are applying for), deanery/administrative staff and potentially a lay member of the public.

Prepare a folder of your academic achievements, this will serve as evidence for your application claims as well as forming the basis of questions during the interview. Having a well laid out folder will boost your confidence and leave a good impression with the interviewers. Practice interview questions with as many people as possible including setting up a mock interview panel with colleagues who are both within and outside your direct academic or clinical area. You may have someone you are working with who has participated in similar interviews in the past, if so seek their advice and feedback. Whilst something may initially sound good in your head going over an answer with someone else may help provide you with additional insight.

Your interview will consist of questions relating to clinical scenarios commonly encountered in the speciality, these are designed to ensure that the candidate has reached the threshold for expected clinical competencies and is safe in a common clinical scenario. Know how such patients may present and how they may be managed. Questions are likely to be academically themed so have an understanding of the evidence base behind clinical management pathways. Identify if the institution you are applying to is renowned for research in a particular field. If there is a seminal piece of research that the institution has published which has changed practice, then mention this as it will show your enthusiasm for research conducted at the department.

## Tip 12. Promote yourself and consider achievements outside of medicine

Do not be shy, sell yourself and highlight your passion for the speciality and research being undertaken within the speciality. Mention any unique talents that you bring to the table as both an academic and a clinician. Do not be afraid to highlight all the great work you have. The ability to promote yourself shows a sense of self-worth and self-esteem. However, be prepared to validate any claims. Do not merely focus on your academic achievement think laterally as other successes or failures in your life may also demonstrate that you possess a specified skill set. Failures should be embraced as a positive element of research as they can help develop resilience. A life outside of medicine reflects a work-life balance and demonstrates a method of coping with stressors, this could be playing musical instrument, sporting activity, charity or volunteering events. Something that you have persisted and moved forward with over the years and pursued alongside your clinical career.

## Conclusions

The top tips outlined above act as a broad guide to follow when considering applying for a clinical academic training. They illustrate the variety of domains a prospective candidate should be considering in order to make their application a successful one. That being said, this is a challenging career pathway with clinicians often juggling several academic commitments around their normal clinical role. The pathway is long with additional time being spent fulfilling academic requirements. However, an academic career is an exciting career option, allowing an individual the freedom to pursue different opportunities outside the clinical setting which can include teaching, management and leadership as well as developing novel research to improve the health and care of patients. Early career clinicians seeking protected time to undertake research to understand disease processes and translate this into improvements in patient care should be encouraged to apply for clinical academic training. It is important to stress that previous experience in a formal academic training post is not always necessary for successful entry into the academic training pathway and therefore applications are encouraged from all suitable applicants.

## Take Home Messages

• The integrated academic training pathway in the UK offers a unique opportunity for trainees to augment clinical training.
• We summarise 12 essential tips which should help future applicants who are considering this pathway.

## Notes On Contributors

MAW is a British Heart Foundation (BHF) Clinical Training Research Fellow working at the Leeds Institute for Cardiovascular and Metabolic Medicine, University of Leeds. He has previously completed a National Institute for Health Research Academic Clinical Fellowship (NIHR ACF) in vascular surgery in the Yorkshire and Humber training deanery and academic foundation training in NHS Scotland. https://orcid.org/0000-0001-5567-9952.

NA is GP ST3 NIHR ACF in the West Midlands and based at the Institute of Applied Health Research, University of Birmingham. He has previously completed academic foundation programme (AFP) training in the West Midlands deanery.

DJAS is a Hon Professor in Vascular Surgery at the University of Leeds and Consultant Vascular and Trauma Surgeon based at the Leeds General Infirmary. He is the current University Lead for undergraduate research engagement and academic foundation training and supports both NIHR ACF and Clinical Lecturers (CL) in Vascular Surgery. He is a past president of the Vascular Society of GB and Ireland and the current President of the UEMS Section and Board of Vascular Surgery.
