# Supplementary material for: Twelve tips for Applying to Clinical Academic Training in the United Kingdom
Source: MedEdPublish (2016). 2019 Mar 13;8:53. [Version 1] doi: 10.15694/mep.2019.000053.1 (PMC10712611; doi:10.15694/mep.2019.000053.1)
Supplement: Peer review history

# Reviewer report: Reviewer response for version 1

**DOI:** 10.21956/mep.19790.r29754

**Published:** 2020-12-15

**Authors:** Michael Drozd

This review has been migrated. The reviewer awarded 5 stars out of 5 I found this article useful when preparing for ACF applications

Reviewer Expertise:

NA

No decision status is available

# Reviewer report: Reviewer response for version 1

**DOI:** 10.21956/mep.19790.r29756

**Published:** 2019-03-30

**Authors:** Sateesh Babu Arja

**Affiliations:** 1 Avalon University School of Medicine

This review has been migrated. The reviewer awarded 4 stars out of 5 Somehow this paper got my attention even though I am not from the UK. The authors have clearly explained the tips for medical students who would like to enter into the academic clinical training. These tips seem to be very useful and were written in such a way to understand easily. The authors have given very interesting tips like participation in journal clubs and mentoring sessions. They also encouraged the undergraduate medical students to participate in research work, presenting at conferences, and seeking prizes at conferences. They also emphasized the fact that students going for membership exams early and pursuing postgraduate degrees. I believe some teaching job positions at universities in the UK might require fellowship from Higher Education Academy, UK. I came to understand how robust and comprehensive these fellowship applications are when I applied for them. Organizations like AMEE and the Academy of Medical Educators (AoME), and IAMSE in case of the USA providing student memberships. It might be a good idea for the undergraduate medical students to be part of AMEE and AoME especially if they are interested to get into academic positions or Academic Clinical Training. Thank you.

Reviewer Expertise:

NA

No decision status is available

# Reviewer report: Reviewer response for version 1

**DOI:** 10.21956/mep.19790.r29755

**Published:** 2019-03-19

**Authors:** Susmita Reddy Karri

**Affiliations:** 1 Topiwala national medical college and BYL nair charitable hospital, mumbai. india

This review has been migrated. The reviewer awarded 4 stars out of 5 An excellently written paper. Each point is explained in a clear and crisp manner. A must read for every applicant. Although out of the scope of this paper, a few lines on what all can an ACF do in his academic time will help young applicants develop more interest in ACF applications. Maybe that could be submitted as a separate article.

Reviewer Expertise:

NA

No decision status is available
